# Supplementary material for: Transient RUNX1 Expression during Early Mesendodermal Differentiation of hESCs Promotes Epithelial to Mesenchymal Transition through TGFB2 Signaling
Source: Stem Cell Reports. 2016 Oct 6;7(5):884–96. doi: 10.1016/j.stemcr.2016.09.006 (PMC5106514; doi:10.1016/j.stemcr.2016.09.006)
Supplement: Document S1. Figures S1 and S2 and Table S1 [file mmc1.pdf]

Stem Cell Reports, Volume *7*

Supplemental Information

Transient RUNX1 Expression during Early Mesendodermal Differentiation of hESCs Promotes Epithelial to Mesenchymal Transition through TGFB2 Signaling

Jennifer J. VanOudenhove, Ricardo Medina, Prachi N. Ghule, Jane B. Lian, Janet L. Stein, Sayyed K. Zaidi, and Gary S. Stein

SUPPLEMENTAL INFORMATION

A.

Pathway Analysis of Up-regulated Genes

Pathway name	pValue
Cell-cell junction organization	1.30E-04
Adherens junctions interactions	3.72E-04
Cell junction organization	4.83E-04
Cell-Cell communication	2.50E-03
Conversion from APC/C:Cdc20 to APC/C:Cdh1 in late anaphase	2.62E-03
POU5F1 (OCT4), SOX2, NANOG activate genes related to proliferation	5.57E-03
RHO GTPases activate IQGAPs	5.96E-03
O-linked glycosylation	9.64E-03
Transcriptional regulation of pluripotent stem cells	1.80E-02
SCF-beta-TrCP mediated degradation of Emi1	1.94E-02

Pathway Analysis of Down-regulated Genes

Pathway name	pValue
Smooth Muscle Contraction	3.44E-08
Muscle contraction	3.10E-07
GRB7 events in ERBB2 signaling	7.89E-04
Recycling of eIF2:GDP	1.39E-03
Role of Abl in Robo-Slit signaling	1.75E-03
Downregulation of ERBB2:ERBB3 signaling	4.15E-03
RHO GTPases Activate ROCKs	6.04E-03
RHO GTPases activate CIT	6.04E-03
TALDO1 deficiency: failed conversion of SH7P, GA3P to Fru(6)P, E4P	6.74E-03
Axon guidance	6.74E-03

B.

E-Cadherin
Epithelial Splicing Regulatory Protein 1
Protocadherin gamma subfamily A, 10
Protocadherin alpha 11
Occludin
Claudin7
Protocadherin beta 5
Cadherin 9, type 2 (T1-cadherin)

Figure S1. Related to Figure 3. (A) Using DAVID (version 6.7) for GO term analysis the top terms upregulated are involved in cell-cell adhesion and junction interactions. (B) A majority of the top upregulated genes are directly related to maintaining an epithelial phenotype.

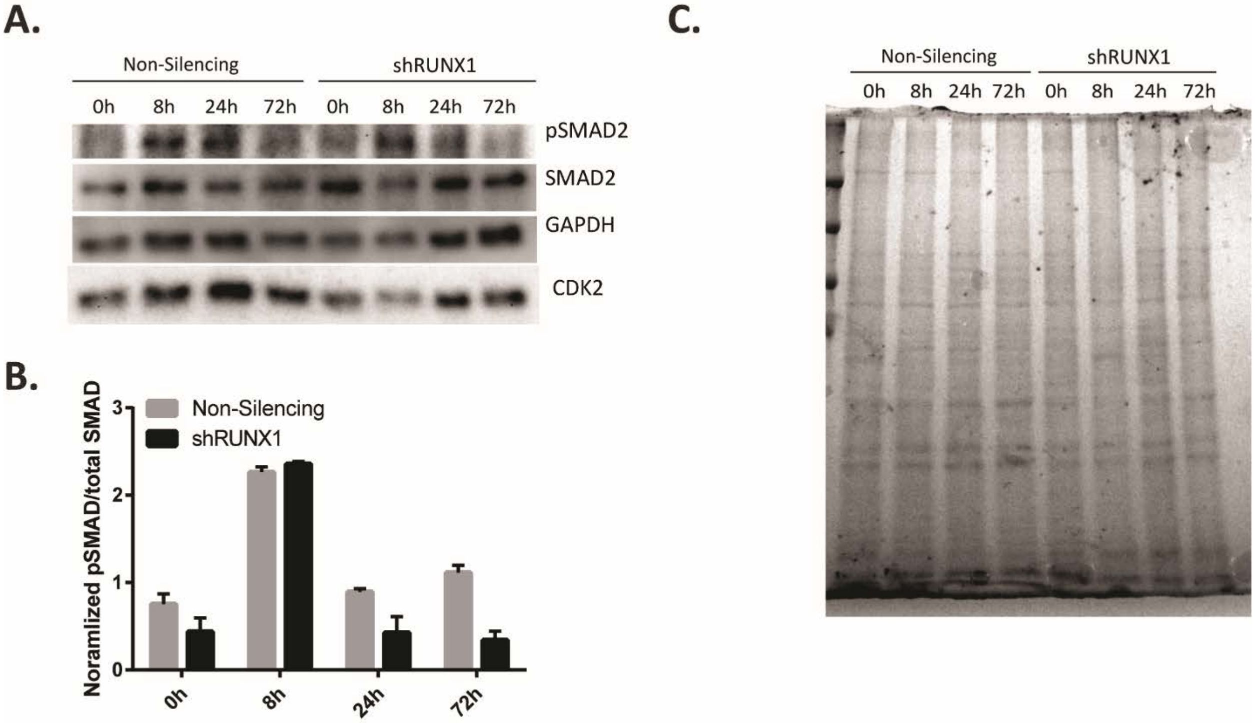

Figure S2. Related to Figure 4. Further Analysis of pSMAD/SMAD2 Western Blot. (A) Representative Western blot showing a decrease in levels of pSMAD2 at 24h and 72h, with an increase in total SMAD2 levels with shRUNX1 treatment during mesendoderm differentiation. Loading controls of GAPDH and CDK2 are included. The blots for pSMAD2 and SMAD2 are the same as those shown in Figure 4D. (B) Quantitation of Western blots from three independent experiments. Data are represented as the mean±SEM of the ratio of pSMAD2 over total SMAD, both normalized using the loading control CDK2. Normalizing to the loading control does not affect the overall result, as there is still a decrease in the ratio of pSMAD to total SMAD at 24h and 72h of differentiation. (C) Coomassie stained gel for Western blot showing no gross total difference in protein loading.

Table S1. Related to Experimental Procedures. qPCR and ChIP primers

Gene for qPCR	Primer	Sequence
PanRUNX1	FW	AGCATGGTGGAGGTGCTG
	REV	GATGGGCAGGGTCTTGTTG
PanRUNX2	FW	ACTTCCTGTGCTCGGTGCT
	REV	CCGGAGCTCAGCAGAATAAT
PanRUNX3	FW	GAGGCTCACTCAGCACCAC
	REV	TCGGAGAATGGGTTCAGTTC
POU5F1	FW	GAGAAGGAGAAGCTGGAGCA
	REV	CTTCTGCTTCAGGAGCTTGG
NANOG	FW	CAGAAGGCCTCAGCACCTAC
	REV	ATTGGAAGGTTCCCAGTCG
T	FW	TGCTTCCCTGAGACCCAGTT
	REV	GATCACTTCTTTCCTTTGCATCAAG
SOX17	FW	AGCAGAATCCAGACCTGCAC
	REV	CTTGTAGTTGGGGTGGTCCT
PAX6	FW	AGACACAGCCCTCACAAACA
	REV	GCAGCATGCAGGAGTATGAG
AFP	FW	AAATGCGTTTCTCGTTGCTT
	REV	GCCACAGGCCAATAGTTTGT
MIXL1	FW	TCCAGGATCCAGGTATGGTT
	REV	AGCCAAAGGTTGGAAGGATT
MYOD1	FW	CCAGGATATGGAGCTACTGTC
	REV	AACACGGGTCGTCATAGAAG
PPARG	FW	CCTATTGACCCAGAAAGCGATT
	REV	CATTACGGAGAGATCCACGGA
HPRT1	FW	GACCAGTCAACAGGGGACAT
	REV	CCTGACCAAGGAAAGCAAAG
RUNX1a	FW	CACATGGGACAATTGTGAGG
	REV	GTACCGGGATCCATGCTAAA
RUNX1b	FW	GGCATGACAACCCTCTCTGC
	REV	GCGTCGGGGAGTAGGTGAA
RUNX1c	FW	GTTTCGCAGCGTGGTAAAAG
	REV	AAGCACTGTGGGTACGAAGG
TGFB1	FW	CGCGTGCTAATGGTGGAAAC
	REV	GTTCAGGTACCGCTTCTCGG
TGFB2	FW	CTTTGGATGCGGCCTATTGC
	REV	TCCAGCACAGAAGTTGGCAT
TGFB3	FW	GGGTCCATGAACCTAAGGGC
	REV	AGGCAGATGCTTCAGGGTTC
CDH1	FW	GGAAGTCAGTTCAGACTCCAGCC
	REV	AGGCCTTTTGACTGTAATCACACC
OCLN	FW	GCAAAGTGAATGACAAGCGGT
	REV	CTGTAACGAGGCTGCCTGAA
CLDN7	FW	CCGAGGAGAGAGCACTTTGG
	REV	TGGATTTCCCTCGAACACCG
VIM	FW	AGGAAATGGCTCGTCACCTTCGTGAATA
	REV	GGAGTGTCGGTTGTTAAGAACTAGAGCT
TWIST1	FW	TGAGCAAGATTCAGACCCTCA
	REV	ATCCTCCAGACCGAGAAGG
ZEB2	FW	AAGCCAGGGACAGATCAGC
	REV	CCACACTCTGTGCATTTGAACT
SNAI 1	FW	CCAATCGGAAGCCTAACTACAG
	REV	GACAGAGTCCCAGATGAGCATT

SNAI2	FW	ACAGCGAACTGGACACACATAC
	REV	GTATCCGGAAAGAGGAGAGAGG
CD44	FW	CCATTTTGCCCTTCCATAGC
	REV	CAACCCCCAACCTCAGTGG
Genes for ChIP	Primer	Sequence
TGFB1	FW	TCTGGGGTTGCCTTCATCTA
	REV	CAGCATTTGGGAAAGGAGAG
TGFB2	FW	AACATAGTGGATCCTGACTGCAA
	REV	TCTGGCAGATGAGGAGACTGA
RUNX1	FW	CTCCCGGGGCCTCTCATC
	REV	CGCCGGGGAGCTTAATTG
