# Supplementary material for: Transient RUNX1 Expression during Early Mesendodermal Differentiation of hESCs Promotes Epithelial to Mesenchymal Transition through TGFB2 Signaling
Source: Stem Cell Reports. 2016 Oct 6;7(5):884–96. doi: 10.1016/j.stemcr.2016.09.006 (PMC5106514; doi:10.1016/j.stemcr.2016.09.006)
Supplement: Document S2. Article plus Supplemental Information [file mmc4.pdf]

Stem Cell Reports
Article
OPEN ACCESS

Transient RUNX1 Expression during Early Mesendodermal Differentiation of hESCs Promotes Epithelial to Mesenchymal Transition through TGFB2 Signaling

Jennifer J. VanOudenhove,[1,2] Ricardo Medina,[2,3] Prachi N. Ghule,[1] Jane B. Lian,[1] Janet L. Stein,[1] Sayyed K. Zaidi,[1] and Gary S. Stein[1,*]
[1]Department of Biochemistry and University of Vermont Cancer Center, University of Vermont College of Medicine, Burlington, VT 05405, USA
[2]Department of Cell and Developmental Biology, University of Massachusetts Medical School, Worcester, MA 01655, USA
[3]Present address: Blueprint Medicines, Cambridge, MA 02139, USA
*Correspondence: gary.stein@uvm.edu
http://dx.doi.org/10.1016/j.stemcr.2016.09.006

SUMMARY

The transition of human embryonic stem cells (hESCs) from pluripotency to lineage commitment is not fully understood, and a role for phenotypic transcription factors in the initial stages of hESC differentiation remains to be explored. From a screen of candidate factors, we found that RUNX1 is selectively and transiently upregulated early in hESC differentiation to mesendodermal lineages. Transcriptome profiling and functional analyses upon RUNX1 depletion established a role for RUNX1 in promoting cell motility. In parallel, we discovered a loss of repression for several epithelial genes, indicating that loss of RUNX1 impaired an epithelial to mesenchymal transition during differentiation. Cell biological and biochemical approaches revealed that RUNX1 depletion specifically compromised TGFB2 signaling. Both the decrease in motility and deregulated epithelial marker expression upon RUNX1 depletion were rescued by reintroduction of TGFB2, but not TGFB1. These findings identify roles for RUNX1-TGFB2 signaling in early events of mesendodermal lineage commitment.

INTRODUCTION

Human embryonic stem cells (hESCs) have unlimited replicative potential and are capable of differentiating into cell types from each of the three germ layers (Thomson et al., 1998). While much is known about the maintenance of pluripotency (Boward et al., 2016; Boyer et al., 2005; Chambers et al., 2003; Huang et al., 2015; Kapinas et al., 2013), how differentiation signals regulate the dissolution of pluripotency and the establishment of phenotype is not well understood. Studies in hESCs have shown that many genes responsible for early developmental events are poised for either activation or repression by epigenetic mechanisms (Bernstein et al., 2006; Grandy et al., 2015; Szutorisz and Dillon, 2005). Once a differentiation signal has been introduced, early factors are expressed that prime the gene expression program of cells for lineage acquisition (Zaret and Carroll, 2011). In this study, we investigated whether phenotype-associated transcription factors may play an initial role in differentiation prior to their established function in specifying lineage identity. A candidate screen of phenotypic transcription factors identified RUNX1 as selectively and transiently upregulated as early as 8 hr during mesendodermal differentiation of hESCs.

Members of the RUNX family of transcription factors have known roles in development (Chuang et al., 2013): RUNX1 is necessary for definitive hematopoiesis (Okuda et al., 1996), RUNX2 for bone formation (Otto et al., 1997), and RUNX3 for gastrointestinal and nervous system development (Inoue et al., 2002; Levanon et al., 2002; Li et al., 2002). When genetically deleted in mice, *Runx1* causes embryonic lethality due to major defects in the formation of the fetal liver and hemorrhaging in the CNS (Okuda et al., 1996; Wang et al., 1996). Emerging evidence suggests that RUNX1 has roles in non-hematopoietic lineages (Osorio et al., 2008; Scheitz and Tumbar, 2013; Stifani et al., 2008). Our discovery of transient upregulation of RUNX1 points to a role for RUNX1 during early mesendodermal differentiation of hESCs.

We investigated what role the early expressed phenotypic transcription factor RUNX1 might play during differentiation in addition to its known role in association with hematopoietic lineage identity. Genome-wide transcriptome analysis of RUNX1-depleted hESCs revealed that RUNX1 positively regulates transforming growth factor β2 (TGFB2) signaling and the motility of the differentiating hESCs. Importantly, genes associated with the epithelial to mesenchymal transition (EMT) were affected, with epithelial gene expression increasing in the absence of RUNX1. Exogenous reintroduction of TGFB2, but not TGFB1, ameliorates the effect of RUNX1 depletion on cell motility and the EMT process. Taken together, our results show that the selective and transient expression of RUNX1 during early hESC differentiation to mesendoderm promotes EMT and motility through regulation of the TGFB2 signaling pathway.

This is an open access article under the CC BY license (http://creativecommons.org/licenses/by/4.0/).

RESULTS

RUNX1 Is Transiently and Selectively Upregulated during Early Mesendodermal Differentiation of Human Embryonic Stem Cells

To investigate whether phenotype-associated transcription factors play a role in early hESC differentiation prior to their established role in control of lineage identity, we screened expression of candidate transcription factors using qRT-PCR analysis. We induced mesendodermal differentiation (Figure 1A) as described in Experimental Procedures, and ensured mesendodermal commitment of hESCs by evaluating the expression of known mesendoderm markers (Mahmood and Aldahmash, 2015; Tada et al., 2005; VanOudenhove et al., 2016). As expected *T*, *MIXL1*, and *MESP1* were upregulated (Figure 1B). We discovered *RUNX1* was the only candidate factor selectively and transiently upregulated as early as 4–8 hr following induction of differentiation (Figure 1C).

Different *RUNX1* isoforms (Figure 1D) are linked to distinct biological processes (Brady et al., 2013; Challen and Goodell, 2010; Ran et al., 2013). Importantly, the *RUNX1c* isoform, transcribed from the P1 promoter, is expressed at the time of emergence of definitive hematopoietic precursors, while *RUNX1b*, transcribed from the P2 promoter, is more widely expressed (Sroczynska et al., 2009). Therefore, we used specific primers to examine relative levels of each *RUNX1* isoform. We discovered that the *RUNX1b* isoform was the predominantly expressed transcript in two different hESC lines, the female H9 and male H1, during mesendodermal differentiation (Figure 1D).

We next examined the levels of total *RUNX1* transcripts during endodermal (D'Amour et al., 2005), mesodermal (Lian et al., 2013), and ectodermal (Tonge and Andrews, 2010) differentiation of hESCs to determine whether expression is lineage specific (Figure 1E); lineages were confirmed using markers as previously described (VanOudenhove et al., 2016). *RUNX1* was expressed during both endodermal and mesodermal, but not ectodermal differentiation, confirming the mesendodermal specificity. Furthermore, RNA FISH (fluorescence in situ hybridization) revealed that >98% of colonies and >95% of cells exhibited *RUNX1* expression during mesendodermal differentiation (Figure 1F). The majority of cells had two nuclear foci, consistent with two sites of transcription. In agreement with the RNA expression data, there was a complete lack of *RUNX1* foci in undifferentiated hESCs (Figure 1F). These findings establish that *RUNX1* transcripts are transiently and selectively expressed in mesendodermal lineage commitment.

Because several post-transcriptional mechanisms can prevent translation (Jackson et al., 2010), we investigated whether the *RUNX1* RNA was translated into protein. Both H1 and H9 hESC lines were subjected to early mesendodermal differentiation. Western blot analysis showed that RUNX1 protein was detectable by 12 hr, peaked at 48 hr, and decreased by 96 hr (Figure 1G). As expected, levels of the pluripotency marker POU5F1 decreased during differentiation (Figure 1G). Since RUNX1 protein is functionally organized in punctate nuclear foci (Zeng et al., 1997, 1998), we investigated its localization using immunofluorescence (IF) microscopy (Figure 1H). Undifferentiated hESCs were devoid of RUNX1 protein, but robust nuclear staining was detected in >90% of cells by 48 hr, which corroborates the RNA expression and FISH results. Taken together, these findings demonstrate that RUNX1 upregulation is specific for early differentiation toward mesendodermal lineages.

RUNX1 Regulates Cell Motility and EMT in Differentiating hESCs

To determine the functional role of RUNX1, we performed global gene expression profiling during early mesendodermal differentiation of hESCs in which RUNX1 had been depleted (Figure 2A). Knockdown of RUNX1 was confirmed by western blot (Figure 2B), and microarray analysis was performed on total cellular RNA from uninfected, non-silencing (shNS) and RUNX1-depleted (shRUNX1) hESCs at four time points (0 hr, 8 hr, 24 hr, 72 hr) (Figure 2A). Reproducibility of gene expression datasets from three independent experiments was demonstrated by principal component analysis, which shows the undifferentiated and differentiated hESC samples in distinct clusters. As differentiation progresses, shRUNX1 samples separate away from the control samples (uninfected and shNS) (Figure 2A). Bioinformatics analyses determined that the non-silencing small hairpin RNA (shRNA) had very little effect on the time course of differentiation. Upon RUNX1 knockdown, only a small number of genes were significantly changed (1.5-fold change with a p value ≤ 0.05 and false discovery rate [FDR] p value ≤ 0.05) by 8 hr of differentiation (n = 31). The number of genes that were changed continued to increase by 24 hr (n = 334) and 72 hr (n = 435) (Figure 2C and Table S2). These observations indicate that the depletion of RUNX1 affects gene expression and suggest a functional role for RUNX1 in early hESC differentiation.

We investigated which biological processes were altered by RUNX1 knockdown during mesendodermal differentiation. Functional grouping of gene ontology (GO) annotations showed that three biological processes were potentially affected: regulation of endothelial cell proliferation, smooth muscle cell migration, and cell adhesion (Figure 2D). We experimentally addressed contributions of RUNX1 in the regulation of these processes. Proliferation was assessed by measuring growth curves of pluripotent or

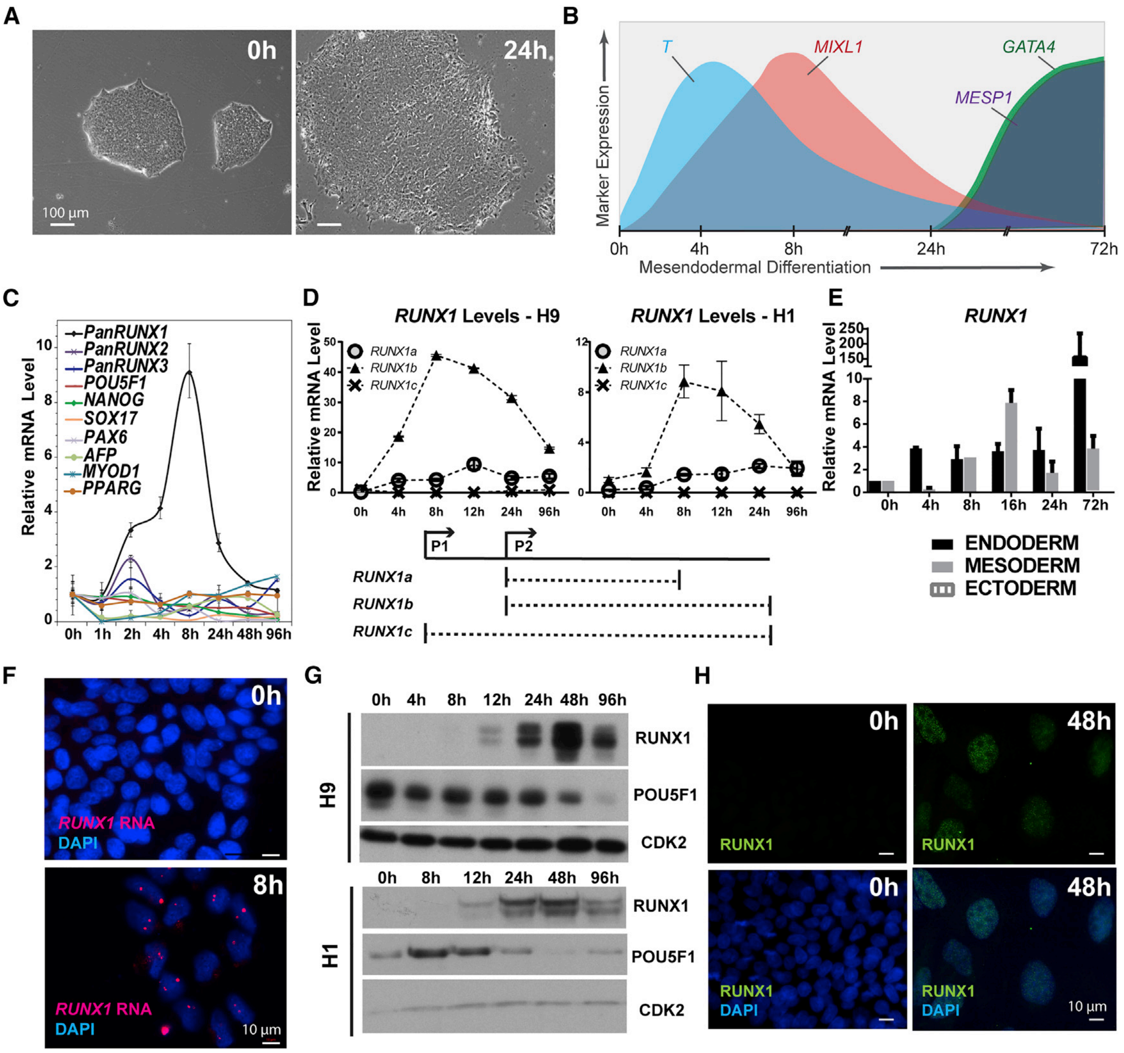

Figure 1. RUNX1 Is Transiently Upregulated during Early Differentiation of Human Embryonic Stem Cells to Mesendodermal Lineages

(A) Phase-contrast images of hESCs before differentiation and after 24 hr of mesendodermal differentiation. In the undifferentiated state the cells appear more epithelial-like, but after 24 hr of differentiation they appear more mesenchymal-like. Images were taken at 10× magnification.

(B) Diagram of gene expression profile of early mesendodermal differentiation of hESCs.

(C) Relative transcript levels of select transcription factors, including the *RUNX* family, during early mesendodermal differentiation of hESCs. Graph represents mean ± SEM from three independent experiments.

(D) Relative levels of the *RUNX1* isoforms in both the female (H9) and male (H1) hESCs with a schematic of different *RUNX1* transcript isoforms. Graph represents mean ± SEM from three independent experiments.

(E) Relative levels of total *RUNX1* transcript during directed differentiation to the three germ layers. No *RUNX1* transcript was detected during ectoderm differentiation. Data represent the mean ± SEM values from three independent experiments.

(F) Representative RNA FISH images showing *RUNX1* RNA (red) at time points indicated. Nuclei are stained with DAPI (blue). Images taken at 63× magnification.

(G) Representative western blot showing the transient increase in levels of RUNX1, and the decrease in POU5F1 in the H1 and H9 hESCs during a mesendoderm differentiation time course with CDK2 used as the loading control.

(H) Representative immunofluorescence images showing RUNX1 (green) nuclear staining at the time points indicated. Nuclei are stained with DAPI (blue). Images taken at 63× magnification.

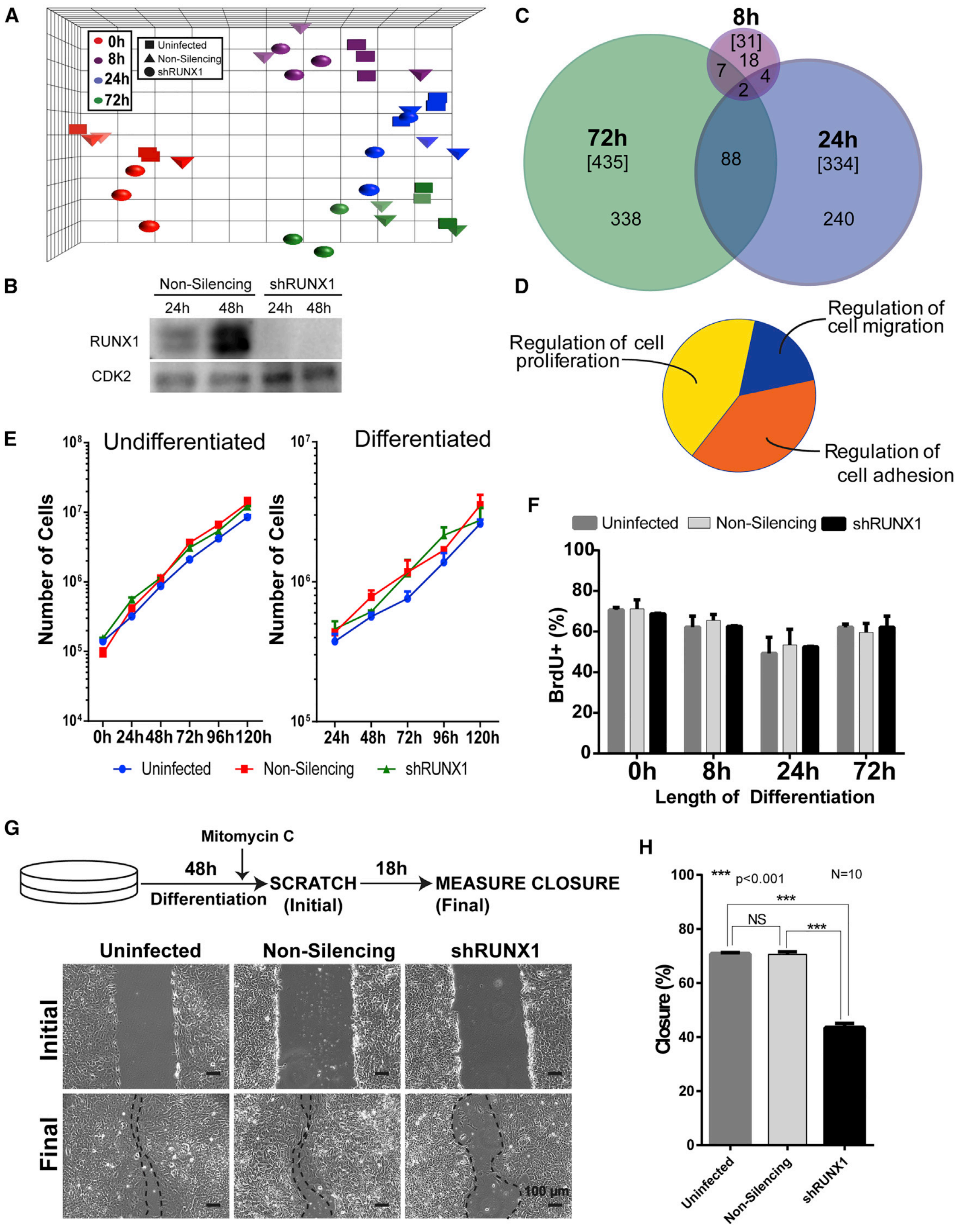

Figure 2. Knockdown of RUNX1 Impairs the Migration Ability, but Not the Proliferation Rate, of hESCs during Mesendoderm Differentiation

(A) Principal component analysis of the time points, replicates, and treatments for mesendodermal differentiation of hESCs from transcriptome profiling. Four time points (undifferentiated [0h; red], 8 hr [8h; purple], 1 day [24h; blue], and 3 days [72h; green]) (n = 3 replicates from independent experiments) were analyzed by microarray analysis under three different treatments (uninfected [squares], non-silencing infected [triangles], and shRUNX1 [circles]).

(legend continued on next page)

differentiating uninfected, shNS, or shRUNX1 hESCs (Figure 2E). RUNX1 depletion had no net effect on proliferation when compared with the two controls. This observation was further confirmed by measuring active DNA synthesis using 5-bromo-2-deoxyuridine (BrdU) incorporation (Figure 2F), which showed no effect of RUNX1 knockdown on the percentage of BrdU- positive cells in pluripotent or differentiating hESCs.

The effect of RUNX1 on the migration of differentiating hESCs was examined by scratch closure assays (Figures 2G and 2H). The extent of scratch closure was measured 18 hr after scratch initiation (Figure 2G). Uninfected and shRNA control cells achieved ~70% closure, while the shRUNX1 cells achieved only ~40% closure (Figure 2H), indicating impaired migration upon RUNX1 depletion. Taken together with the lack of RUNX1 effect on proliferation, these findings establish a role for RUNX1 in regulating migration during early mesendodermal differentiation.

Consistent with these results, GO term analysis using DAVID revealed that genes upregulated upon knockdown of RUNX1 were involved in cell-cell adhesion and junction interactions (Figure S1A). In addition, many of the top genes that were increased upon RUNX1 depletion during differentiation are associated with an epithelial-like phenotype (Figure S1B). These findings led us to experimentally test whether the EMT was affected by loss of RUNX1. We evaluated the expression of several epithelial (*CDH1* [Cadherin 1/E-Cadherin], *OCLN* [Occludin], and *CLD7* [Claudin 7]) and mesenchymal (*VIM* [Vimentin], *TWIST1* [Twist Family basic Helix-Loop-Helix Transcription Factor 1], *ZEB2* [Zinc Finger E-box Binding Homeobox 2], *SNAI1* [Snail Family Transcriptional Repressor 1], *SNAI2* [Snail Family Transcriptional Repressor 2], and *CD44* [CD44 molecule (Indian Blood Group)]) marker genes across early mesendodermal differentiation in either shNS or shRUNX1 hESCs (Figure 3). A typical profile of cells undergoing EMT is observed in shNS hESCs, where all epithelial markers decreased and all mesenchymal markers increased at some point during differentiation (Figure 3, light gray bars). However, in the absence of RUNX1, the epithelial markers decreased initially but were restored to at least undifferentiated levels by 72 hr (Figure 3, black bars). This is consistent with RUNX1, which is expressed by 8 hr, assisting in the suppression of epithelial markers during the early stages of EMT. In contrast, the majority of mesenchymal markers were unaffected with the exception of *ZEB2*, which was not induced (Figure 3, black bars). Collectively these findings show that RUNX1 contributes to regulation of a physiological EMT that occurs during early mesendodermal differentiation.

RUNX1 Depletion Inhibits TGFB2 Signaling

Because EMT is a complex process regulated by multiple signaling pathways (Derynck et al., 2014; Zhang et al., 2016), we performed signaling pathway analysis on annotated genes that changed significantly upon RUNX1 depletion (Figure 4A and Table S3). Distinct pathways were suggested to be activated by RUNX1 knockdown at each time point, although no single pathway was suggested to be activated at all time points. Conversely, the TGFB pathway was indicated to be the most inhibited across the time course of differentiation (Figure 4A and Table S3), which suggests that RUNX1 activates TGFB signaling during mesendodermal differentiation.

To investigate directly how RUNX1 regulates the TGFB signaling pathway, we examined expression of the *TGFB* ligands upon RUNX1 depletion (Figure 4B). *TGFB1* and *TGFB2* RNA exhibited sequential and significant upregulation (>15 fold) during hESC differentiation, while *TGFB3*, which is expressed at relatively low levels, remained

(B) Representative western blot comparing the levels of RUNX1 in hESCs treated with either non-silencing shRNA or RUNX1 shRNA differentiating to mesendoderm, confirming that RUNX1 is knocked down in shRUNX1 hESCs.
(C) Venn diagram of the number of genes with expression changes greater than 1.5-fold, and p value and FDR p values less than 0.05, at each differentiation time point under shRUNX1 treatment compared with non-silencing infected hESCs. The total number of genes changed at each time point is in brackets.
(D) ClueGO analysis of genes with significant expression changes reveals three biological processes that might be affected by RUNX1 knockdown.
(E) Growth curves for hESCs either uninfected (blue), non-silencing infected (red), or with shRUNX1 (green) under pluripotent and mesendoderm differentiation conditions. Line graph represents mean ± SEM from three independent experiments.
(F) Percentage of cells staining positive for BrdU with a 30-min pulse of labeling. Quantification of $BrdU^+$ cells was performed using blind scoring in duplicate of 200 cells from immunofluorescent images. Data represent mean ± SEM from three independent experiments.
(G) Representative phase-contrast images from a scratch closure assay. Cells were plated, differentiated for 48 hr, and after 46 hr of differentiation cells were treated with mitomycin C (to inhibit proliferation) after which a scratch was made. Closure was measured 18 hr later. Dotted lines have been added to the phase-contrast images for emphasis along the edge of the scratch after that 18 h period. All phase-contrast images were taken at 10× magnification.
(H) Percentage of scratch closure for hESCs uninfected, non-silencing infected, and infected with shRUNX1, as quantitated by ImageJ plug-in. Ten scratches were measured for each condition, two from each of five independent experiments, and data represent mean ± SD. ***$p < 0.001$; NS, not significant.

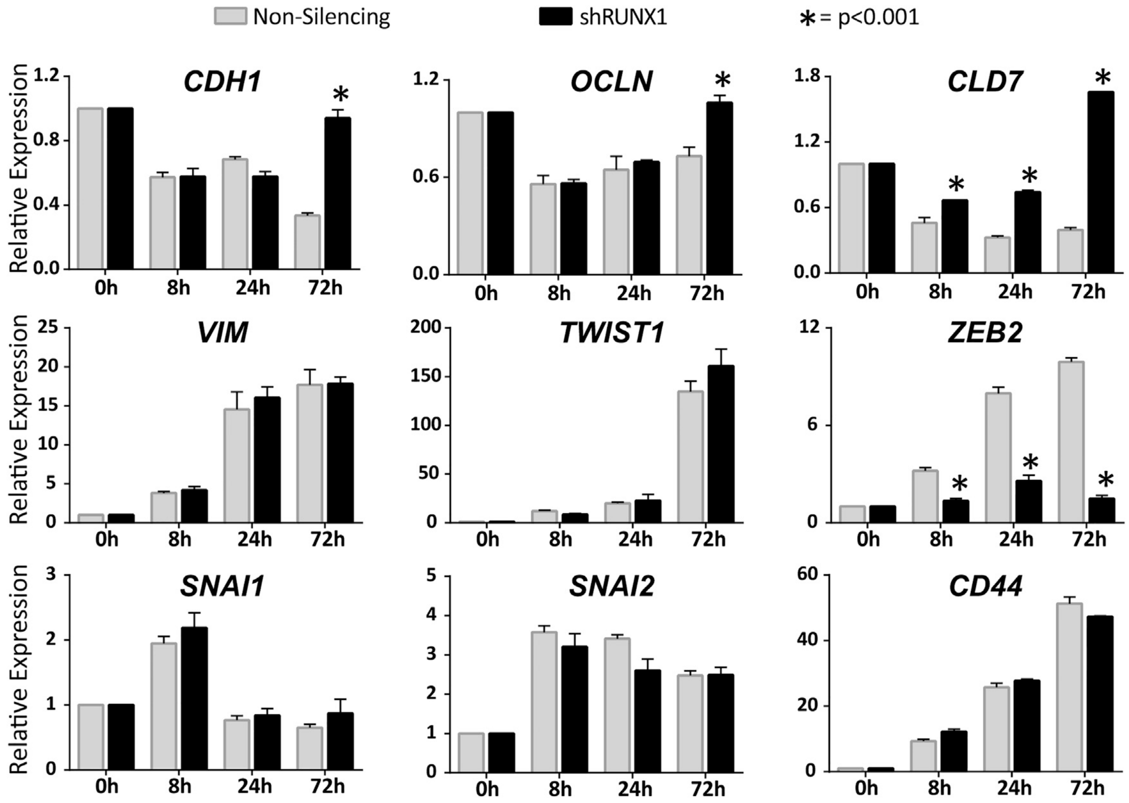

Figure 3. Genes that Regulate Epithelial to Mesenchymal Transition Are Affected by RUNX1 Knockdown

During mesendoderm differentiation of hESCs, the levels of multiple effectors of EMT (epithelial associated [*CDH1*, *OCLN*, and *CLD7*] and mesenchymal associated [*VIM*, *TWIST1*, *ZEB2*, *SNAI1*, *SNAI2*, and *CD44*]) were measured by qRT-PCR under treatment by non-silencing infection, or shRUNX1. During the control (non-silencing) differentiation epithelial marker expression decreases and mesenchymal gene expression increases. However, with shRUNX1 treatment, the epithelial marker genes fail to be suppressed and *ZEB2* is not induced. Data shown represent mean ± SEM from three independent experiments. p Values were determined by t test between non-silencing infected and shRUNX1 treatment at each corresponding time point (*p < 0.001). See also Figure S1.

unchanged. Importantly, only *TGFB2* expression was significantly affected by RUNX1 depletion (Figure 4B). We also found that both *TGFB1* and *TGFB2*, but not *TGFB3*, promoters contain RUNX1 consensus sites within 1 kb of the transcription start site. We tested whether RUNX1 binds to the *TGFB* promoters using chromatin immunoprecipitation (ChIP)-qPCR. Our results show that RUNX1 selectively occupies the *TGFB2*, but not the *TGFB1*, promoter (Figure 4C), which is consistent with the effect of RUNX1 depletion on *TGFB2* expression (Figure 4B). As expected, RUNX1 occupied its own promoter, which was included as a positive control (Knezevic et al., 2011). These results show that RUNX1 selectively occupies and regulates expression of the *TGFB2* gene.

We next examined whether downstream effectors of the canonical TGFB pathway were changed in the absence of RUNX1. Western blot analysis was carried out for both phospho- and total SMAD2 during mesendodermal differentiation (Figures 4D and S2). In the control (non-silencing) time course, there was an increase in phospho-SMAD2 at 8 hr and 24 hr that decreased by 72 hr relative to total SMAD2 (Figures 4D, S2A, and S2B). This increase coincides with the expression of *TGFB1* and *TGFB2* ligands that both signal through the SMAD pathway (Figures 4B, 4D, and S2). Quantitation of the ratio of phospho- to total SMAD2 revealed a decrease at 24 hr and 72 hr, which is when *TGFB2* levels are decreased the most upon RUNX1 knockdown (Figures 4D, 4E, and S2B). Together, these findings show that RUNX1 is an upstream activator of TGFB2 signaling during early mesendodermal differentiation.

TGFB2 Rescues Impaired Cell Motility and Epithelial Gene Expression Caused by RUNX1 Depletion

The TGFB pathway is known to regulate cell migration and adhesion (Xu et al., 2009). Our findings revealed that RUNX1 affects cell motility (Figure 2G) and specifically regulates *TGFB2* expression (Figure 4B). We directly tested whether there is a connection between the defects in

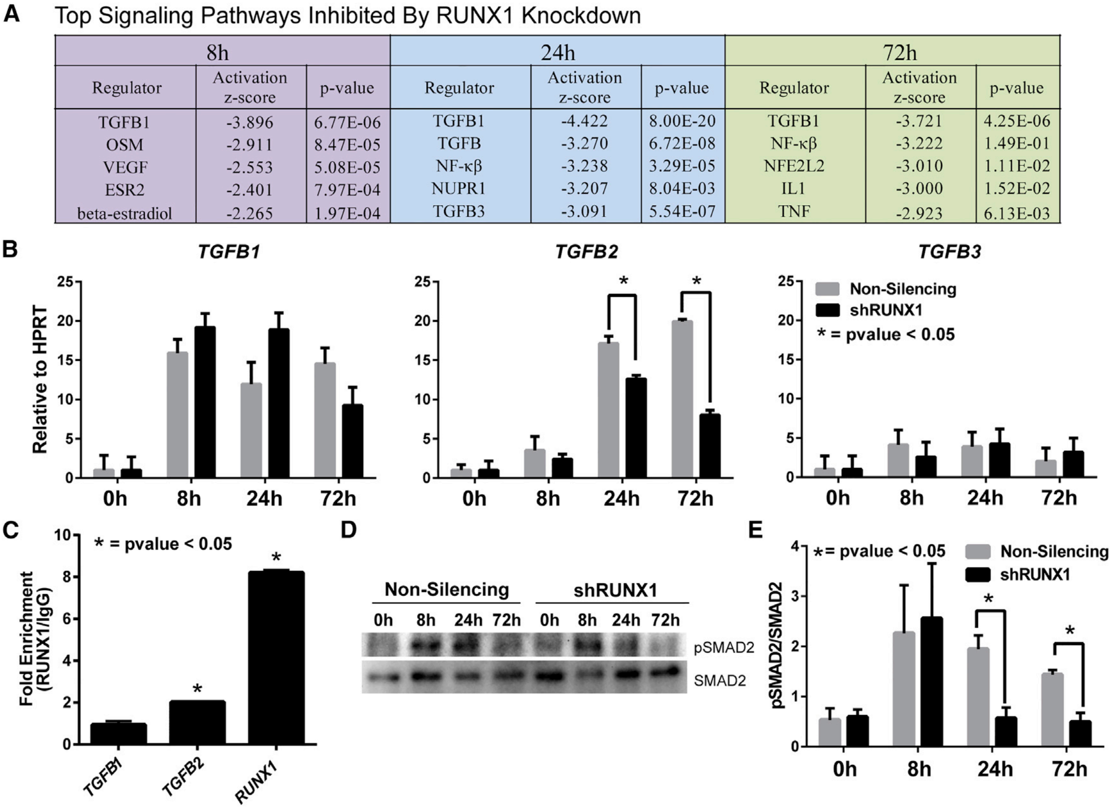

A Top Signaling Pathways Inhibited By RUNX1 Knockdown

8h			24h			72h		
Regulator	Activation z-score	p-value	Regulator	Activation z-score	p-value	Regulator	Activation z-score	p-value
TGFB1	-3.896	6.77E-06	TGFB1	-4.422	8.00E-20	TGFB1	-3.721	4.25E-06
OSM	-2.911	8.47E-05	TGFB	-3.270	6.72E-08	NF-κβ	-3.222	1.49E-01
VEGF	-2.553	5.08E-05	NF-κβ	-3.238	3.29E-05	NFE2L2	-3.010	1.11E-02
ESR2	-2.401	7.97E-04	NUPR1	-3.207	8.04E-03	IL1	-3.000	1.52E-02
beta-estradiol	-2.265	1.97E-04	TGFB3	-3.091	5.54E-07	TNF	-2.923	6.13E-03

Figure 4. Knockdown of RUNX1 Inhibits the TGFB Signaling Pathway, Specifically through *TGFB2*

(A) Top signaling pathways inhibited by RUNX1 knockdown as determined by Ingenuity Pathway analysis.

(B) Relative expression of the three *TGFB* ligands under non-silencing and shRUNX1 treatment by RT-qPCR. Data represent mean ± SEM from three independent experiments with a p value determined by t test (*p < 0.05).

(C) ChIP-qPCR analysis for RUNX1 binding sites in the promoters of the *TGFB1*, *TGFB2*, and *RUNX1* promoters. Experiments were carried out at the peak of RUNX1 protein expression at 48 hr of differentiation. Data represent mean ± SEM of fold enrichment from four independent experiments with a p value determined by t test comparing specific signaling with signal obtained from the IgG control (*p < 0.05).

(D) Representative western blot showing a decrease in levels of pSMAD2 at 24 and 72 hr, with an increase in total SMAD2 levels with shRUNX1 treatment during mesendoderm differentiation.

(E) Quantitation of western blots from four independent experiments. Data represent the mean ± SEM of the ratio of pSMAD2 over total SMAD2, and there is a significant decrease (*p < 0.05) in the ratio of pSMAD2 to total SMAD2 at 24 and 72 hr of differentiation.

See also Figure S2.

motility and EMT gene expression that result from RUNX1 depletion and TGFB2 signaling. Scratch closure assays were performed to determine whether the effect of RUNX1 depletion on hESC migration could be rescued by TGFB2 (Figure 5A). We found that addition of TGFB2 significantly increased the extent of scratch closure of the shRUNX1 hESCs from ~40% to ~60%; TGFB1, which was included as a control, had no effect (Figure 5B). These findings are consistent with our discovery that RUNX1 occupies and transcriptionally activates the *TGFB2* gene (Figures 4B and 4C), and establish that RUNX1 regulation of hESC migration is mediated through TGFB2.

TGFB signaling can regulate EMT and the associated changes in cell adhesion and migration (Nieto, 2013; Thiery et al., 2009). Because RUNX1 knockdown inhibits TGFB signaling (Figure 4), we evaluated whether supplementation of the shRUNX1 cultures with exogenous TGFB1 or TGFB2 could rescue the altered expression of EMT genes (Figure 5C). While TGFB2 caused significant repression of the epithelial markers to levels similar to those in shNS hESCs, TGFB1 was unable to repress most of these genes, with the exception of *CLDN7* (Figure 5C). TGFB2 failed to induce expression of *ZEB2*, the only mesenchymal marker that was not upregulated during differentiation of shRUNX1 hESCs, suggesting that the effect of TGFB2 is primarily on epithelial markers (Figure 5C). These findings indicate that knockdown of RUNX1 alleviates repression of epithelial genes (Figure 3), and addition

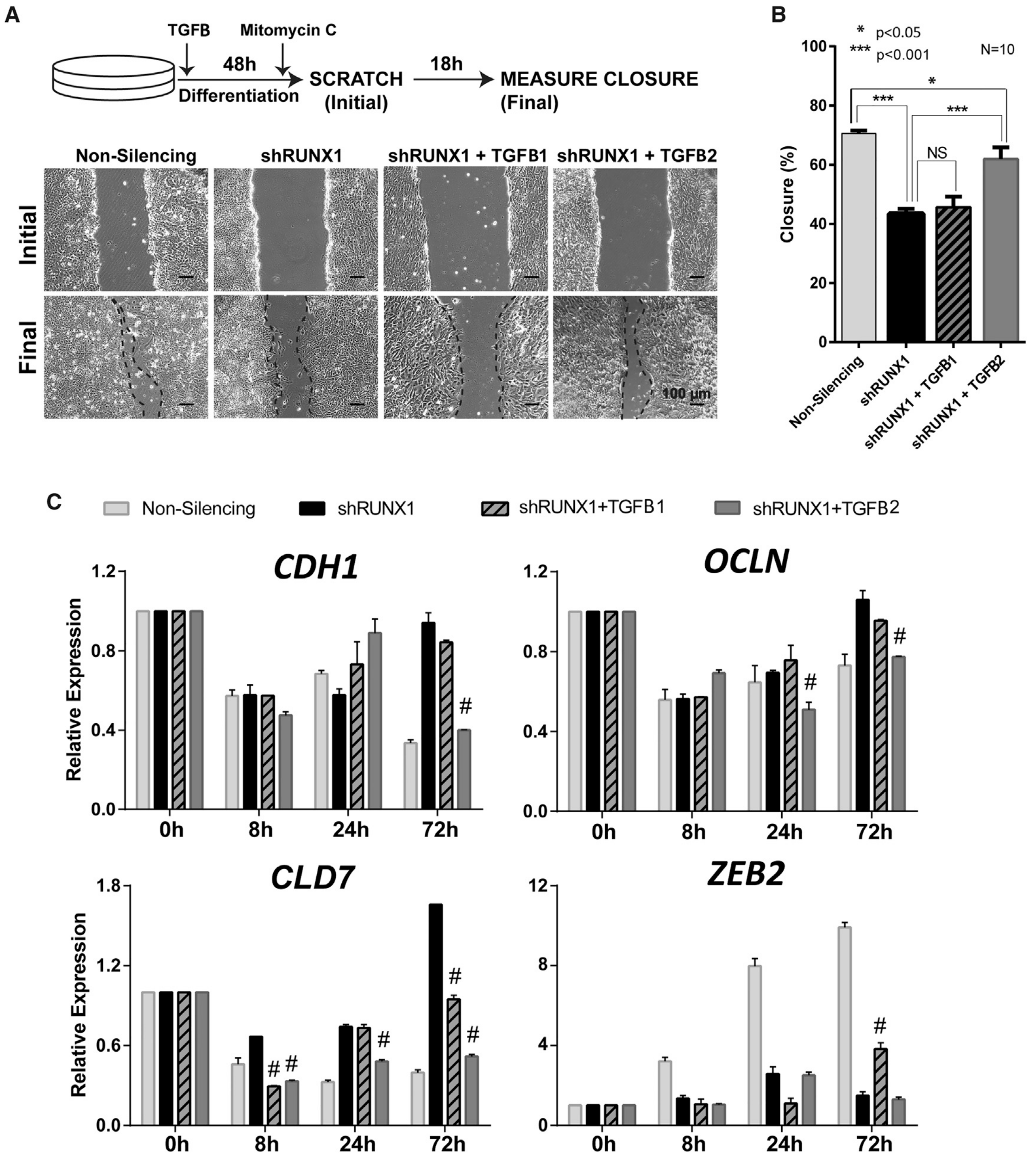

Figure 5. Defects in Cell Motility and EMT Gene Expression Caused by RUNX1 Depletion Are Rescued by Reintroduction of TGFB2, but Not TGFB1

(A) Representative phase-contrast images from a scratch closure assay. Assays were carried out as in Figure 2G, with the addition of TGFB ligand. Dotted lines have been added to the phase-contrast images for emphasis along the edge of the scratch after the 18 h closure period. All phase-contrast images were taken at 10× magnification.

(B) Percentage of scratch closure for hESCs with non-silencing infection and shRUNX1 infection, as well as shRUNX1 cells supplemented with exogenous TGFB1 or TGFB2, as quantitated by ImageJ. Ten scratches were measured for each condition, two from each of five independent experiments, and data represent mean ± SD. p Values were determined by t test (*$p < 0.05$, ***$p < 0.001$).

(C) The expression of the four genes that were found to be affected by RUNX1 knockdown (*CDH1*, *OCLN*, *CLD7*, and *ZEB2*) with non-silencing, shRUNX1, shRUNX1 + TGFB2, and shRUNX1 + TGFB1 over a mesendoderm differentiation time course. Data are shown as mean ± SEM from three independent experiments. p Values were determined by t test between shRUNX1 and either shRUNX1 cells supplemented with TGFB2, or TGFB1 ($^{\#}p < 0.0001$).

of TGFB2 decreases the expression of these epithelial genes (Figure 5C). Taken together, our results show that the selective and transient RUNX1 expression during early mesendodermal differentiation of hESCs regulates cell motility and EMT gene expression through TGFB2.

DISCUSSION

In this study we discovered unexpected, transient expression of the phenotypic transcription factor RUNX1 during early mesendodermal differentiation of hESCs, which suggested that RUNX1 contributes to differentiation in addition to its established role in hematopoietic lineage identity. Our findings showed that RUNX1 regulates cell motility and gene expression during mesendodermal differentiation, specifically through TGFB2 signaling. These results reveal a previously unknown role for RUNX1 in early development.

The discovery of a burst of *RUNX1* expression was both selective and specific because none of the other examined phenotypic transcription factors showed increased expression. *RUNX1* expression from the distal P1 promoter is linked with the emergence of definitive hematopoietic stem cells (Chen et al., 2009; Lacaud et al., 2002; Okuda et al., 1996), but little work has been done on the role of transcripts from the more ubiquitous proximal P2 promoter (Challen and Goodell, 2010; Fujita et al., 2001; Sroczynska et al., 2009). In this study, the *RUNX1* transcript that we found during early mesendodermal differentiation is the *RUNX1b* isoform from the P2 promoter. Of note, in undifferentiated hESCs the *RUNX1* P2 promoter that expresses the RUNX1b transcript is bivalently marked with H3K27me3 and H3K4me3, indicating that it is poised for expression, while the P1 promoter is not (Mikkelsen et al., 2007). Our discovery of a rapid, but substantial increase in RUNX1 expression early in differentiation to mesendoderm suggests a potential role for RUNX1 that is unrelated to hematopoiesis (Lian et al., 2003).

We provide evidence that RUNX1 has a role in early mesendodermal differentiation of hESCs through regulation of cell migration and adhesion, as indicated by impairment of these processes upon RUNX1 depletion. These findings are consistent with the emerging role of RUNX1 in controlling cell motility and migration in other biological systems. Our laboratory has previously shown that Runx1 depletion in breast cancer cells results in a decreased migration and invasion phenotype (Browne et al., 2015); similar results were found in ovarian cancer cells (Keita et al., 2013). Likewise, Runx1b is responsible for inducing a cell adhesion and migration program prior to release of mouse hematopoietic stem cells from hemogenic endothelium (Lie-A-Ling et al., 2014).

Gene expression profiling of early mesendodermal differentiation revealed that RUNX1 regulates TGFB signaling, which has known roles in the maintenance of pluripotency of hESCs (James et al., 2005; Wei et al., 2005), differentiation (Itoh et al., 2014; Watabe and Miyazono, 2009), and EMT (Xu et al., 2009). The inhibition of motility and derepression of epithelial genes observed upon RUNX1 depletion indicates that RUNX1 is upstream of the TGFB pathway. Moreover, we found that RUNX1 specifically occupies and regulates the expression of the *TGFB2* gene, which encodes one of the three TGFB ligands. Although the TGFB ligands share greater than 70% homology (Kingsley, 1994), studies of knockout mice show non-overlapping phenotypes, indicating that each ligand has specific functions in development (Sanford et al., 1997). Consistent with the results from these mouse models, we found that TGFB2, but not TGFB1, rescued the phenotype of RUNX1 depletion.

In conclusion, our discovery of RUNX1-mediated regulation of TGFB2 signaling provides mechanistic insights into early mesendodermal differentiation. These results also establish RUNX1 as a selective and specific regulator of cell motility and EMT-associated gene expression.

EXPERIMENTAL PROCEDURES

Stem Cell Culture

The female H9 (WA09) line and the male H1 (WA01) of hESCs were maintained on Matrigel and differentiated as previously reported (VanOudenhove et al., 2016). In short, mesendoderm differentiation was induced with Knockout DMEM, containing 20% heat-inactivated defined fetal bovine serum (FBS), 1 mM L-glutamine with 1% (v/v) 2-mercaptoethanol, and 0.1 mM non-essential amino acids. Retinoic acid (RA)-induced ectodermal differentiation was induced by the addition of 1 μM all-*trans* retinoic acid (Sigma-Aldrich). The mesodermal differentiation protocol induces differentiation by introducing medium containing RPMI 1640 with B-27 supplement without insulin (Life Technologies) and 12 μM CHIR99021 (Selleck Chemicals, catalog #S2924). For the production of endoderm, undifferentiated hESC cultures were switched to RPMI 1640 containing 1× Glutamax and 100 ng/mL activin A (R&D Systems); for treatment past 24 hr, 0.2% FBS was added.

Lentiviral RNAi

H9 hESCs plated on Matrigel were transduced in 6-well plates with lentivirus carrying shRNA designed to knock down RUNX1 (clone V2LHS_150257) or be non-silencing (#RHS4346) using the GIPZ Lentiviral RNAi System (GE Dharmacon) in the presence of polybrene. After introduction of lentivirus, cells were spun at 2,000 rpm at 37°C for 45 min. Selection with 1 μg/mL puromycin (Sigma-Aldrich #P7255-100MG) was performed for the first three passages after infection.

Microarray Expression Analysis

RNA was extracted using TRIzol and prepared for microarray analysis as described previously (VanOudenhove et al., 2016). All target

preparation and microarray hybridization/scanning was performed in the VGN Microarray Facility at the University of Vermont. The datasets generated have been deposited in the NCBI GEO database according to MIAME guidelines (GEO: GSE74004 and GSE79598).

Due to evident fold-change compression, Affymetrix used a GC content leveling and signal-space transformation to reduce background levels. Analysis of data was performed using Affymetrix Expression Console Build 1.3.1.187 and the Affymetrix Transcriptome Analysis Console Version 1.0.0.234. Differential gene expression was defined as a fold change greater than 1.5, an ANOVA p value less than 0.05, and an FDR p value less than 0.05. Partek Genomic Suite software was used to generate the principal component analysis. EulerAPE version 3.0.0 was used to generate the proportional Venn diagram and then recolored (Micallef and Rodgers, 2014). Pathway analysis was performed using Ingenuity Pathways Analysis (Qiagen, www.ingenuity.com). GO term analysis was performed using DAVID (Version 6.7) (Huang da et al., 2009a, 2009b). The ClueGO plug-in for Cytoscape 2.8 (Version 1.8) was used for functional grouping GO analysis (Bindea et al., 2009).

Real-Time qPCR Analysis

RNA was isolated as described for microarray analysis, and cDNA was synthesized with random hexamer primers using SuperScript III First Strand Synthesis System (Life Technologies #18080-051). qRT-PCR was performed using SYBR Green PCR Master Mix (Bio-Rad); samples were normalized to *HPRT1* and fold change was determined using the ΔΔCt method. Primers used are as specified in Table S1.

Immunofluorescence Microscopy and RNA FISH

Cells were grown on Matrigel-coated coverslips for IF and RNA FISH. Detection of RUNX1 protein was performed using a rabbit polyclonal RUNX1 antibody (Cell Signaling #4336). Staining was performed using a fluorescent secondary antibody to rabbit polyclonal antibodies and a goat anti-rabbit immunoglobulin G (IgG) (H + L) secondary antibody, Alexa Fluor 488 conjugate (Thermo Fisher Scientific #A-11001). For RNA FISH, a *RUNX1* probe was created using a BAC clone (RP11-299D9) spanning the *RUNX1* gene locus, obtained from the BAC/PAC Resources at the Children's Hospital Oakland Research Institute from the RPCI-11 Human Male BAC Library (Osoegawa et al., 1998, 2001). The BAC clone was amplified and isolated using the Qiagen Large-Construct Kit, and labeled by nick translation using the DIG-Nick Translation Mix (Roche #11745816910). Hybridization and detection were carried out as previously reported (Byron et al., 2013), with the addition of a pepsin digest (5 mg/mL pepsin in a 1:2,000 dilution in 0.01 N HCl) to allow full penetrance of probe. Hybridization occurred overnight at 37°C. A rhodamine anti-digoxygenin secondary antibody (Roche #11207750910) was used for signal detection. Images were taken on a Zeiss AxioImager microscope equipped with a Hamamatsu CCD camera and Metamorph imaging software.

Proliferation Assays

For growth curves, cells were plated in 12-well plates. The next day, cells were counted and this value was taken as 0 hr, and differentiation was initiated in half of the plates. Counting was performed at the same time daily for up to 120 hr until confluence was reached. For the BrdU incorporation assay, cells were incubated for 30 min at 37°C with 10 μM BrdU (Roche kit #11296 736 001) to allow for incorporation before fixation. Fixation was performed using 3.7% formaldehyde in PBS for 10 min. Cells were then permeabilized in 0.1% Triton X-100 in PBS, and washed in 0.5% BSA in PBS. For the BrdU incorporation assay, cells were treated with DNaseI (30 μg per million cells) (BD Biosciences) for 1 hr at 37°C after permeabilization to expose the incorporated BrdU. Detection was performed using a mouse monoclonal anti-BrdU antibody (clone MBG 6H8 IgG1 from Roche) and staining was performed using a mouse monoclonal F(ab′)2 goat anti-mouse IgG (H + L) secondary antibody and Alexa Fluor 647 conjugate (Life Technologies #A-21237).

Western Blot

Whole-cell lysates were generated by incubating cells in RIPA buffer for 30 min on ice, followed by sonication using a Covaris S-220 Ultrasonic Processor for 5 min. Lysates were separated in an 8% polyacrylamide gel and transferred to polyvinylidene fluoride membranes (Millipore) using an OWL semi-dry transfer apparatus. Membranes were blocked using 1% Blotting Grade Blocker Non-Fat Dry Milk (Bio-Rad) and incubated overnight at 4°C with the following primary antibodies: a rabbit polyclonal RUNX1 (Cell Signaling #4334, 1:1,000); a goat polyclonal to POU5F1 (Santa Cruz Biotechnology #sc-8628, 1:1,000); a rabbit polyclonal to CDK2 (M2) (Santa Cruz #sc-163, 1:2,000); a mouse monoclonal to GAPDH (0411) (Santa Cruz #sc-47724); a rabbit monoclonal to SMAD2 (D43B4) (Cell Signaling #5339); and a rabbit monoclonal to pSMAD2 (Ser465/467) (138D4) (Cell Signaling #3108). Secondary antibodies conjugated to horseradish peroxidase (Santa Cruz) were used for immunodetection, along with the Clarity Western ECL Substrate (Bio-Rad) on a Chemidoc XRS+ imaging system (Bio-Rad).

TGFB Rescues

Reintroduction of TGFB1 (R&D Systems #240-B) and TGFB2 (R&D Systems #302-B2) reconstituted in 4 mM HCl was performed at 5.0 ng/mL.

Scratch Assays

hESCs were plated and 24 hr later were induced to differentiate, and 30 hr into differentiation mitomycin C at 10 μg/mL was introduced to inhibit cell proliferation. After 48 hr of differentiation, colonies were scratched down the center using a sterile 10-μL pipette tip. Scratches were imaged by phase-contrast microscopy directly in the center of the colony, after being washed twice with medium, and marked. The scratch was imaged 18 hr later in the marked location. The scratch area was calculated using the Scratch Assay Analyzer plug-in from MiToBo toolbox for ImageJ (Glaß et al., 2012).

Chromatin Immunoprecipitation

After 2 days of mesendodermal differentiation, cells were cross-linked with 0.8% formaldehyde (Sigma-Aldrich) in PBS for 10 min. The cells were washed twice and lysed in a buffer containing 0.1% sodium deoxycholate, 0.5% N-lauroylsarcosine, 1 mM

EDTA, 1 mM EGTA, 10 mM Tris-HCl (pH 8), and 1× protease inhibitor mixture. Lysates were sonicated to fragment chromatin DNA into ~200–800 bp pieces using an S220 Sonicator (Covaris). Lysates were transferred into a buffer containing 0.1% SDS, 1 mM EDTA, 50 mM HEPES (pH 7.9), 140 mM NaCl, 1% Triton X-100, and 1× protease inhibitor mixture. Sheared chromatin was used for immunoprecipitation with RUNX1 antibody (Cell Signaling #4334) or IgG (Millipore #12-370) overnight at 4°C. Subsequently, the immunoprecipitation mixture was incubated with Protein-G Dynabeads (Thermo Fisher #10003D) for an additional 3 hr at 4°C. Precipitated chromatin was washed with salt solutions of increasing concentration, and eluted into a buffer containing 1% SDS and 0.1 M $NaHCO_3$. The crosslinking was reversed and DNA was recovered using the QiaQuick PCR purification kit (Qiagen #28104).

ACCESSION NUMBERS

The accession number for the new data reported in this paper is GEO: GSE79598.

SUPPLEMENTAL INFORMATION

Supplemental Information includes two figures and three tables and can be found with this article online at http://dx.doi.org/10.1016/j.stemcr.2016.09.006.

AUTHOR CONTRIBUTIONS

J.J.V.: Conception and design, collection/assembly of data, data analysis and interpretation, manuscript writing, final approval of manuscript. R.M.: Conception and design, collection/assembly of data. P.N.G.: Conception and design, collection/assembly of data, data analysis and interpretation. J.B.L.: Data analysis and interpretation. J.L.S.: Conception and design, assembly of data, data analysis and interpretation, financial support, manuscript writing, final approval of manuscript. S.K.Z.: Conception and design, assembly of data, data analysis and interpretation, manuscript writing. G.S.S.: Conception and design, data analysis and interpretation, financial support, manuscript writing, final approval of manuscript.

ACKNOWLEDGMENTS

We thank the members of our laboratory, as well as Julie Dragon of the UVM Bioinformatics Shared Resource for her help with microarray quality control. This work was supported by NIH grants NCI P01 CA082834 (G.S.S., J.L.S.), R01 CA139322 (G.S.S.), R37 DE012528 (J.B.L.), and NIGMS IDeA grant P20 GM103449.

Received: May 10, 2016
Revised: September 8, 2016
Accepted: September 9, 2016
Published: October 6, 2016

REFERENCES

Bernstein, B.E., Mikkelsen, T.S., Xie, X., Kamal, M., Huebert, D.J., Cuff, J., Fry, B., Meissner, A., Wernig, M., Plath, K., et al. (2006). A bivalent chromatin structure marks key developmental genes in embryonic stem cells. Cell *125*, 315–326.

Bindea, G., Mlecnik, B., Hackl, H., Charoentong, P., Tosolini, M., Kirilovsky, A., Fridman, W.H., Pages, F., Trajanoski, Z., and Galon, J. (2009). ClueGO: a Cytoscape plug-in to decipher functionally grouped gene ontology and pathway annotation networks. Bioinformatics *25*, 1091–1093.

Boward, B., Wu, T., and Dalton, S. (2016). Concise review: control of cell fate through cell cycle and pluripotency networks. Stem Cells *34*, 1427–1436.

Boyer, L.A., Lee, T.I., Cole, M.F., Johnstone, S.E., Levine, S.S., Zucker, J.P., Guenther, M.G., Kumar, R.M., Murray, H.L., Jenner, R.G., et al. (2005). Core transcriptional regulatory circuitry in human embryonic stem cells. Cell *122*, 947–956.

Brady, G., Elgueta Karstegl, C., and Farrell, P.J. (2013). Novel function of the unique N-terminal region of RUNX1c in B cell growth regulation. Nucleic Acids Res. *41*, 1555–1568.

Browne, G., Taipaleenmaki, H., Bishop, N.M., Madasu, S.C., Shaw, L.M., van Wijnen, A.J., Stein, J.L., Stein, G.S., and Lian, J.B. (2015). Runx1 is associated with breast cancer progression in MMTV-PyMT transgenic mice and its depletion in vitro inhibits migration and invasion. J. Cell. Physiol. *230*, 2522–2532.

Byron, M., Hall, L.L., and Lawrence, J.B. (2013). A multifaceted FISH approach to study endogenous RNAs and DNAs in native nuclear and cell structures. Curr. Protoc. Hum. Genet. *Chapter 4*, Unit 4.15.

Challen, G.A., and Goodell, M.A. (2010). Runx1 isoforms show differential expression patterns during hematopoietic development but have similar functional effects in adult hematopoietic stem cells. Exp. Hematol. *38*, 403–416.

Chambers, I., Colby, D., Robertson, M., Nichols, J., Lee, S., Tweedie, S., and Smith, A. (2003). Functional expression cloning of Nanog, a pluripotency sustaining factor in embryonic stem cells. Cell *113*, 643–655.

Chen, M.J., Yokomizo, T., Zeigler, B.M., Dzierzak, E., and Speck, N.A. (2009). Runx1 is required for the endothelial to haematopoietic cell transition but not thereafter. Nature *457*, 887–891.

Chuang, L.S., Ito, K., and Ito, Y. (2013). RUNX family: regulation and diversification of roles through interacting proteins. Int. J. Cancer *132*, 1260–1271.

D'Amour, K.A., Agulnick, A.D., Eliazer, S., Kelly, O.G., Kroon, E., and Baetge, E.E. (2005). Efficient differentiation of human embryonic stem cells to definitive endoderm. Nat. Biotechnol. *23*, 1534–1541.

Derynck, R., Muthusamy, B.P., and Saeteurn, K.Y. (2014). Signaling pathway cooperation in TGF-beta-induced epithelial-mesenchymal transition. Curr. Opin. Cell Biol. *31*, 56–66.

Fujita, Y., Nishimura, M., Taniwaki, M., Abe, T., and Okuda, T. (2001). Identification of an alternatively spliced form of the mouse AML1/RUNX1 gene transcript AML1c and its expression in early hematopoietic development. Biochem. Biophys. Res. Commun. *281*, 1248–1255.

Glaß, M., Möller, B., Zirkel, A., Wächter, K., Hüttelmaier, S., and Posch, S. (2012). Cell migration analysis: segmenting scratch assay

images with level sets and support vector machines. Pattern Recogn. *45*, 3154–3165.

Grandy, R.A., Whitfield, T.W., Wu, H., Fitzgerald, M.P., VanOudenhove, J.J., Zaidi, S.K., Montecino, M.A., Lian, J.B., van Wijnen, A.J., Stein, J.L., et al. (2015). Genome-wide studies reveal that H3K4me3 modification in bivalent genes is dynamically regulated during the pluripotent cell cycle and stabilized upon differentiation. Mol. Cell. Biol. *36*, 615–627.

Huang da, W., Sherman, B.T., and Lempicki, R.A. (2009a). Bioinformatics enrichment tools: paths toward the comprehensive functional analysis of large gene lists. Nucleic Acids Res. *37*, 1–13.

Huang da, W., Sherman, B.T., and Lempicki, R.A. (2009b). Systematic and integrative analysis of large gene lists using DAVID bioinformatics resources. Nat. Protoc. *4*, 44–57.

Huang, G., Ye, S., Zhou, X., Liu, D., and Ying, Q.L. (2015). Molecular basis of embryonic stem cell self-re from signaling pathways to pluripotency network. Cell. Mol. Life Sci. *72*, 1741–1757.

Inoue, K., Ozaki, S., Shiga, T., Ito, K., Masuda, T., Okado, N., Iseda, T., Kawaguchi, S., Ogawa, M., Bae, S.C., et al. (2002). Runx3 controls the axonal projection of proprioceptive dorsal root ganglion neurons. Nat. Neurosci. *5*, 946–954.

Itoh, F., Watabe, T., and Miyazono, K. (2014). Roles of TGF-beta family signals in the fate determination of pluripotent stem cells. Semin. Cell Dev. Biol. *32*, 98–106.

Jackson, R.J., Hellen, C.U., and Pestova, T.V. (2010). The mechanism of eukaryotic translation initiation and principles of its regulation. Nat. Rev. Mol. Cell Biol. *11*, 113–127.

James, D., Levine, A.J., Besser, D., and Hemmati-Brivanlou, A. (2005). TGFbeta/activin/nodal signaling is necessary for the maintenance of pluripotency in human embryonic stem cells. Development *132*, 1273–1282.

Kapinas, K., Grandy, R., Ghule, P., Medina, R., Becker, K., Pardee, A., Zaidi, S.K., Lian, J., Stein, J., van Wijnen, A., et al. (2013). The abbreviated pluripotent cell cycle. J. Cell. Physiol. *228*, 9–20.

Keita, M., Bachvarova, M., Morin, C., Plante, M., Gregoire, J., Renaud, M.C., Sebastianelli, A., Trinh, X.B., and Bachvarov, D. (2013). The RUNX1 transcription factor is expressed in serous epithelial ovarian carcinoma and contributes to cell proliferation, migration and invasion. Cell Cycle *12*, 972–986.

Kingsley, D.M. (1994). The TGF-beta superfamily: new members, new receptors, and new genetic tests of function in different organisms. Genes Dev. *8*, 133–146.

Knezevic, K., Bee, T., Wilson, N.K., Janes, M.E., Kinston, S., Polderdijk, S., Kolb-Kokocinski, A., Ottersbach, K., Pencovich, N., Groner, Y., et al. (2011). A Runx1-Smad6 rheostat controls Runx1 activity during embryonic hematopoiesis. Mol. Cell. Biol. *31*, 2817–2826.

Lacaud, G., Gore, L., Kennedy, M., Kouskoff, V., Kingsley, P., Hogan, C., Carlsson, L., Speck, N., Palis, J., and Keller, G. (2002). Runx1 is essential for hematopoietic commitment at the hemangioblast stage of development in vitro. Blood *100*, 458–466.

Levanon, D., Bettoun, D., Harris-Cerruti, C., Woolf, E., Negreanu, V., Eilam, R., Bernstein, Y., Goldenberg, D., Xiao, C., Fliegauf, M., et al. (2002). The Runx3 transcription factor regulates development and survival of TrkC dorsal root ganglia neurons. EMBO J. *21*, 3454–3463.

Li, Q.L., Ito, K., Sakakura, C., Fukamachi, H., Inoue, K., Chi, X.Z., Lee, K.Y., Nomura, S., Lee, C.W., Han, S.B., et al. (2002). Causal relationship between the loss of RUNX3 expression and gastric cancer. Cell *109*, 113–124.

Lian, J.B., Balint, E., Javed, A., Drissi, H., Vitti, R., Quinlan, E.J., Zhang, L., van Wijnen, A.J., Stein, J.L., Speck, N., et al. (2003). Runx1/AML1 hematopoietic transcription factor contributes to skeletal development in vivo. J. Cell. Physiol. *196*, 301–311.

Lian, X., Zhang, J., Azarin, S.M., Zhu, K., Hazeltine, L.B., Bao, X., Hsiao, C., Kamp, T.J., and Palecek, S.P. (2013). Directed cardiomyocyte differentiation from human pluripotent stem cells by modulating Wnt/beta-catenin signaling under fully defined conditions. Nat. Protoc. *8*, 162–175.

Lie-A-Ling, M., Marinopoulou, E., Li, Y., Patel, R., Stefanska, M., Bonifer, C., Miller, C., Kouskoff, V., and Lacaud, G. (2014). RUNX1 positively regulates a cell adhesion and migration program in murine hemogenic endothelium prior to blood emergence. Blood *124*, e11–e20.

Mahmood, A., and Aldahmash, A. (2015). Induction of primitive streak and mesendoderm formation in monolayer hESC culture by activation of TGF-beta signaling pathway by Activin B. Saudi J. Biol. Sci. *22*, 692–697.

Micallef, L., and Rodgers, P. (2014). eulerAPE: drawing area-proportional 3-Venn diagrams using ellipses. PLoS One *9*, e101717.

Mikkelsen, T.S., Ku, M., Jaffe, D.B., Issac, B., Lieberman, E., Giannoukos, G., Alvarez, P., Brockman, W., Kim, T.K., Koche, R.P., et al. (2007). Genome-wide maps of chromatin state in pluripotent and lineage-committed cells. Nature *448*, 553–560.

Nieto, M.A. (2013). Epithelial plasticity: a common theme in embryonic and cancer cells. Science *342*, 1234850.

Okuda, T., van Deursen, J., Hiebert, S.W., Grosveld, G., and Downing, J.R. (1996). AML1, the target of multiple chromosomal translocations in human leukemia, is essential for normal fetal liver hematopoiesis. Cell *84*, 321–330.

Osoegawa, K., Woon, P.Y., Zhao, B., Frengen, E., Tateno, M., Catanese, J.J., and de Jong, P.J. (1998). An improved approach for construction of bacterial artificial chromosome libraries. Genomics *52*, 1–8.

Osoegawa, K., Mammoser, A.G., Wu, C., Frengen, E., Zeng, C., Catanese, J.J., and de Jong, P.J. (2001). A bacterial artificial chromosome library for sequencing the complete human genome. Genome Res. *11*, 483–496.

Osorio, K.M., Lee, S.E., McDermitt, D.J., Waghmare, S.K., Zhang, Y.V., Woo, H.N., and Tumbar, T. (2008). Runx1 modulates developmental, but not injury-driven, hair follicle stem cell activation. Development *135*, 1059–1068.

Otto, F., Thornell, A.P., Crompton, T., Denzel, A., Gilmour, K.C., Rosewell, I.R., Stamp, G.W., Beddington, R.S., Mundlos, S., Olsen, B.R., et al. (1997). Cbfa1, a candidate gene for cleidocranial dysplasia syndrome, is essential for osteoblast differentiation and bone development. Cell *89*, 765–771.

Ran, D., Shia, W.J., Lo, M.C., Fan, J.B., Knorr, D.A., Ferrell, P.I., Ye, Z., Yan, M., Cheng, L., Kaufman, D.S., et al. (2013). RUNX1a enhances hematopoietic lineage commitment from human

embryonic stem cells and inducible pluripotent stem cells. Blood *121*, 2882–2890.

Sanford, L.P., Ormsby, I., Gittenberger-de Groot, A.C., Sariola, H., Friedman, R., Boivin, G.P., Cardell, E.L., and Doetschman, T. (1997). TGFbeta2 knockout mice have multiple developmental defects that are non-overlapping with other TGFbeta knockout phenotypes. Development *124*, 2659–2670.

Scheitz, C.J., and Tumbar, T. (2013). New insights into the role of Runx1 in epithelial stem cell biology and pathology. J. Cell. Biochem. *114*, 985–993.

Sroczynska, P., Lancrin, C., Kouskoff, V., and Lacaud, G. (2009). The differential activities of Runx1 promoters define milestones during embryonic hematopoiesis. Blood *114*, 5279–5289.

Stifani, N., Freitas, A.R., Liakhovitskaia, A., Medvinsky, A., Kania, A., and Stifani, S. (2008). Suppression of interneuron programs and maintenance of selected spinal motor neuron fates by the transcription factor AML1/Runx1. Proc. Natl. Acad. Sci. USA *105*, 6451–6456.

Szutorisz, H., and Dillon, N. (2005). The epigenetic basis for embryonic stem cell pluripotency. Bioessays *27*, 1286–1293.

Tada, S., Era, T., Furusawa, C., Sakurai, H., Nishikawa, S., Kinoshita, M., Nakao, K., Chiba, T., and Nishikawa, S. (2005). Characterization of mesendoderm: a diverging point of the definitive endoderm and mesoderm in embryonic stem cell differentiation culture. Development *132*, 4363–4374.

Thiery, J.P., Acloque, H., Huang, R.Y., and Nieto, M.A. (2009). Epithelial-mesenchymal transitions in development and disease. Cell *139*, 871–890.

Thomson, J.A., Itskovitz-Eldor, J., Shapiro, S.S., Waknitz, M.A., Swiergiel, J.J., Marshall, V.S., and Jones, J.M. (1998). Embryonic stem cell lines derived from human blastocysts. Science *282*, 1145–1147.

Tonge, P.D., and Andrews, P.W. (2010). Retinoic acid directs neuronal differentiation of human pluripotent stem cell lines in a non-cell-autonomous manner. Differentiation *80*, 20–30.

VanOudenhove, J.J., Grandy, R.A., Ghule, P.N., Del Rio, R., Lian, J.B., Stein, J.L., Zaidi, S.K., and Stein, G.S. (2016). Lineage-specific early differentiation of human embryonic stem cells requires a G2 cell cycle pause. Stem Cells *34*, 1765–1775.

Wang, Q., Stacy, T., Binder, M., Marin-Padilla, M., Sharpe, A.H., and Speck, N.A. (1996). Disruption of the Cbfa2 gene causes necrosis and hemorrhaging in the central nervous system and blocks definitive hematopoiesis. Proc. Natl. Acad. Sci. USA *93*, 3444–3449.

Watabe, T., and Miyazono, K. (2009). Roles of TGF-beta family signaling in stem cell renewal and differentiation. Cell Res. *19*, 103–115.

Wei, C.L., Miura, T., Robson, P., Lim, S.K., Xu, X.Q., Lee, M.Y., Gupta, S., Stanton, L., Luo, Y., Schmitt, J., et al. (2005). Transcriptome profiling of human and murine ESCs identifies divergent paths required to maintain the stem cell state. Stem Cells *23*, 166–185.

Xu, J., Lamouille, S., and Derynck, R. (2009). TGF-beta-induced epithelial to mesenchymal transition. Cell Res. *19*, 156–172.

Zaret, K.S., and Carroll, J.S. (2011). Pioneer transcription factors: establishing competence for gene expression. Genes Dev. *25*, 2227–2241.

Zeng, C., van Wijnen, A.J., Stein, J.L., Meyers, S., Sun, W., Shopland, L., Lawrence, J.B., Penman, S., Lian, J.B., Stein, G.S., et al. (1997). Identification of a nuclear matrix targeting signal in the leukemia and bone-related AML/CBF-alpha transcription factors. Proc. Natl. Acad. Sci. USA *94*, 6746–6751.

Zeng, C., McNeil, S., Pockwinse, S., Nickerson, J., Shopland, L., Lawrence, J.B., Penman, S., Hiebert, S., Lian, J.B., van Wijnen, A.J., et al. (1998). Intranuclear targeting of AML/CBFalpha regulatory factors to nuclear matrix-associated transcriptional domains. Proc. Natl. Acad. Sci. USA *95*, 1585–1589.

Zhang, J., Tian, X.J., and Xing, J. (2016). Signal transduction pathways of EMT induced by TGF-beta, SHH, and WNT and their crosstalks. J. Clin. Med. *5*, 41.

Stem Cell Reports, Volume *7*

Supplemental Information

Transient RUNX1 Expression during Early Mesendodermal Differentiation of hESCs Promotes Epithelial to Mesenchymal Transition through TGFB2 Signaling

Jennifer J. VanOudenhove, Ricardo Medina, Prachi N. Ghule, Jane B. Lian, Janet L. Stein, Sayyed K. Zaidi, and Gary S. Stein

SUPPLEMENTAL INFORMATION

A.

Pathway Analysis of Up-regulated Genes

Pathway name	pValue
Cell-cell junction organization	1.30E-04
Adherens junctions interactions	3.72E-04
Cell junction organization	4.83E-04
Cell-Cell communication	2.50E-03
Conversion from APC/C:Cdc20 to APC/C:Cdh1 in late anaphase	2.62E-03
POU5F1 (OCT4), SOX2, NANOG activate genes related to proliferation	5.57E-03
RHO GTPases activate IQGAPs	5.96E-03
O-linked glycosylation	9.64E-03
Transcriptional regulation of pluripotent stem cells	1.80E-02
SCF-beta-TrCP mediated degradation of Emi1	1.94E-02

Pathway Analysis of Down-regulated Genes

Pathway name	pValue
Smooth Muscle Contraction	3.44E-08
Muscle contraction	3.10E-07
GRB7 events in ERBB2 signaling	7.89E-04
Recycling of eIF2:GDP	1.39E-03
Role of Abl in Robo-Slit signaling	1.75E-03
Downregulation of ERBB2:ERBB3 signaling	4.15E-03
RHO GTPases Activate ROCKs	6.04E-03
RHO GTPases activate CIT	6.04E-03
TALDO1 deficiency: failed conversion of SH7P, GA3P to Fru(6)P, E4P	6.74E-03
Axon guidance	6.74E-03

B.

E-Cadherin
Epithelial Splicing Regulatory Protein 1
Protocadherin gamma subfamily A, 10
Protocadherin alpha 11
Occludin
Claudin7
Protocadherin beta 5
Cadherin 9, type 2 (T1-cadherin)

Figure S1. Related to Figure 3. (A) Using DAVID (version 6.7) for GO term analysis the top terms upregulated are involved in cell-cell adhesion and junction interactions. (B) A majority of the top upregulated genes are directly related to maintaining an epithelial phenotype.

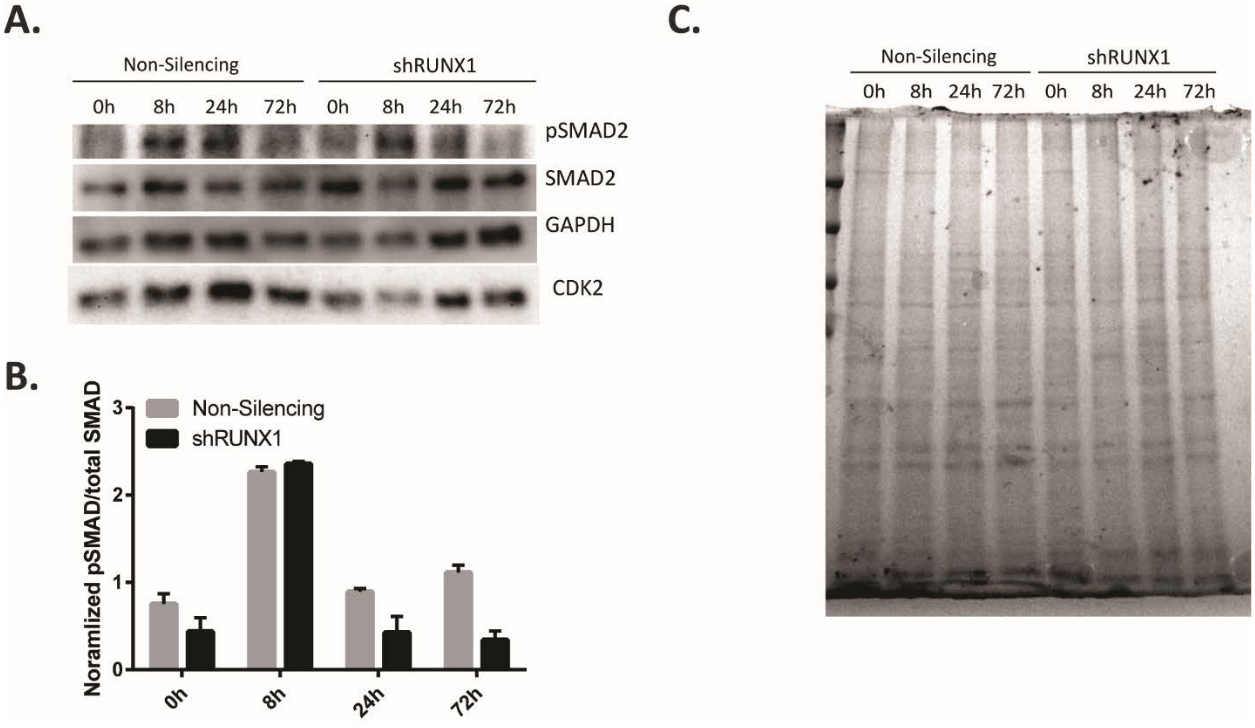

Figure S2. Related to Figure 4. Further Analysis of pSMAD/SMAD2 Western Blot. (A) Representative Western blot showing a decrease in levels of pSMAD2 at 24h and 72h, with an increase in total SMAD2 levels with shRUNX1 treatment during mesendoderm differentiation. Loading controls of GAPDH and CDK2 are included. The blots for pSMAD2 and SMAD2 are the same as those shown in Figure 4D. (B) Quantitation of Western blots from three independent experiments. Data are represented as the mean±SEM of the ratio of pSMAD2 over total SMAD, both normalized using the loading control CDK2. Normalizing to the loading control does not affect the overall result, as there is still a decrease in the ratio of pSMAD to total SMAD at 24h and 72h of differentiation. (C) Coomassie stained gel for Western blot showing no gross total difference in protein loading.

Table S1. Related to Experimental Procedures. qPCR and ChIP primers

Gene for qPCR	Primer	Sequence
PanRUNX1	FW	AGCATGGTGGAGGTGCTG
	REV	GATGGGCAGGGTCTTGTTG
PanRUNX2	FW	ACTTCCTGTGCTCGGTGCT
	REV	CCGGAGCTCAGCAGAATAAT
PanRUNX3	FW	GAGGCTCACTCAGCACCAC
	REV	TCGGAGAATGGGTTCAGTTC
POU5F1	FW	GAGAAGGAGAAGCTGGAGCA
	REV	CTTCTGCTTCAGGAGCTTGG
NANOG	FW	CAGAAGGCCTCAGCACCTAC
	REV	ATTGGAAGGTTCCCAGTCG
T	FW	TGCTTCCCTGAGACCCAGTT
	REV	GATCACTTCTTTCCTTTGCATCAAG
SOX17	FW	AGCAGAATCCAGACCTGCAC
	REV	CTTGTAGTTGGGGTGGTCCT
PAX6	FW	AGACACAGCCCTCACAAACA
	REV	GCAGCATGCAGGAGTATGAG
AFP	FW	AAATGCGTTTCTCGTTGCTT
	REV	GCCACAGGCCAATAGTTTGT
MIXL1	FW	TCCAGGATCCAGGTATGGTT
	REV	AGCCAAAGGTTGGAAGGATT
MYOD1	FW	CCAGGATATGGAGCTACTGTC
	REV	AACACGGGTCGTCATAGAAG
PPARG	FW	CCTATTGACCCAGAAAGCGATT
	REV	CATTACGGAGAGATCCACGGA
HPRT1	FW	GACCAGTCAACAGGGGACAT
	REV	CCTGACCAAGGAAAGCAAAG
RUNX1a	FW	CACATGGGACAATTGTGAGG
	REV	GTACCGGGATCCATGCTAAA
RUNX1b	FW	GGCATGACAACCCTCTCTGC
	REV	GCGTCGGGGAGTAGGTGAA
RUNX1c	FW	GTTTCGCAGCGTGGTAAAAG
	REV	AAGCACTGTGGGTACGAAGG
TGFB1	FW	CGCGTGCTAATGGTGGAAAC
	REV	GTTCAGGTACCGCTTCTCGG
TGFB2	FW	CTTTGGATGCGGCCTATTGC
	REV	TCCAGCACAGAAGTTGGCAT
TGFB3	FW	GGGTCCATGAACCTAAGGGC
	REV	AGGCAGATGCTTCAGGGTTC
CDH1	FW	GGAAGTCAGTTCAGACTCCAGCC
	REV	AGGCCTTTTGACTGTAATCACACC
OCLN	FW	GCAAAGTGAATGACAAGCGGT
	REV	CTGTAACGAGGCTGCCTGAA
CLDN7	FW	CCGAGGAGAGAGCACTTTGG
	REV	TGGATTTCCCTCGAACACCG
VIM	FW	AGGAAATGGCTCGTCACCTTCGTGAATA
	REV	GGAGTGTCGGTTGTTAAGAACTAGAGCT
TWIST1	FW	TGAGCAAGATTCAGACCCTCA
	REV	ATCCTCCAGACCGAGAAGG
ZEB2	FW	AAGCCAGGGACAGATCAGC
	REV	CCACACTCTGTGCATTTGAACT
SNAI 1	FW	CCAATCGGAAGCCTAACTACAG
	REV	GACAGAGTCCCAGATGAGCATT

SNAI2	FW	ACAGCGAACTGGACACACATAC
	REV	GTATCCGGAAAGAGGAGAGAGG
CD44	FW	CCATTTTGCCCTTCCATAGC
	REV	CAACCCCCAACCTCAGTGG
Genes for ChIP	Primer	Sequence
TGFB1	FW	TCTGGGGTTGCCTTCATCTA
	REV	CAGCATTTGGGAAAGGAGAG
TGFB2	FW	AACATAGTGGATCCTGACTGCAA
	REV	TCTGGCAGATGAGGAGACTGA
RUNX1	FW	CTCCCGGGGCCTCTCATC
	REV	CGCCGGGGAGCTTAATTG
